# Determinants of first line antiretroviral therapy treatment failure among adult patients on ART at central Ethiopia: un-matched case control study

**DOI:** 10.1186/s12879-019-4651-6

**Published:** 2019-12-03

**Authors:** Diriba Mulisa, Mulugeta Tesfa, Getachew Mullu Kassa, Tadesse Tolossa

**Affiliations:** 1 School of Nursing and Midwifery, Wollega University, P.O.BOX: 395, Nekemte, Ethiopia; 2 Department of Midwifery, College of Health Science, Debre Markos University, Debre Markos, Ethiopia

## Abstract

**Background:**

In 2018 in Ethiopia, magnitude of human immunodeficiency virus Acquired Immunodeficiency Syndrome treatment failure was 15.9% and currently the number of patient receiving second line antiretroviral therapy (ART) is more increasing than those taking first line ART. Little is known about the predictors of treatment failure in the study area. Therefore; more factors that can be risk for first line ART failure have to identified to make the patients stay on first line ART for long times. Consequently, the aim of this study was to identify determinants of first line ART treatment failure among patients on ART at St. Luke referral hospital and Tulubolo General Hospital, 2019.

**Methods:**

A 1:2 un-matched case-control study was conducted among adult patients on active follow up. One new group variables was formed as group 1 for cases and group 0 for controls and then data was entered in to Epi data version 3 and exported to STATA SE version 14 for analysis. From binary logistic regression variables with *p* value ≤0.25 were a candidate for multiple logistic regression. At the end variables with a *p*-value ≤0.05 were considered as statistically significant.

**Result:**

A total of 350 (117 cases and 233 controls) patients were participated in the study. Starting ART after 2 years of being confirmed HIV positive (AOR = 3.82 95% CI 1.37,10.6), nevirapine (NVP) based initial ART (AOR = 2.77,95%CI 1.22,6.28) having history of lost to follow up (AOR 3.66,95%CI 1.44,9.27) and base line opportunistic infection (AOR = 1.97,95%CI 1.06,3.63), staying on first line ART for greater than 5 years (AOR = 3.42,95%CI 1.63,7.19) and CD4 less than100cell/ul (AOR = 2.72,95%CI 1.46,5.07) were independent determinants of first line ART treatment failure.

**Conclusion:**

Lost to follow up, staying on first line ART for greater than 5 years, presence of opportunistic infections, NVP based NNRT, late initiation of ART are determinant factors for first line ART treatment failure. The concerned bodies have to focus and act on those identified factors to maintain the patient on first line ART.

## Background

Antiretroviral therapy (ART) treatment for human immunodeficiency virus /Acquired Immunodeficiency Syndrome HIV/AIDS started in 1987 [1]. ART treatment failure is associated with virologic failure, immunologic failure, and/or clinical failure.

ART decrease rate of death from HIV/AIDS but as a world, treatment failure is become common problem [1]. As systematic review and meta-analysis conducted in Sub-Saharan Africa indicated the magnitude of first line ART treatment failure is 16% [2]. Multicenter retrospective follow-up study conducted in northwest Ethiopia also indicated that the incidence of first line ART treatment failure is 61.7 per 1000 person years of observation [3].

In Ethiopia, fee based ART was started in 2003 and subsequently in 2005 free ART program was initiated [4, 5]. According to Ethiopian 2008 ART guide line optimum time to initiate ART is when a patient’s CD4 count is 200–350, stage III with CD4 < 350 and stage IV without CD4 consideration [6]. Once-daily regimens comprising g NRTI backbone (TDF + 3TC) and one NNRTI (EFV) are maintained as the preferred choices in adults, adolescents and children older than 10 years [4]. Preferred first Line regimens drugs are TDF + 3TC + EFV (FDC) while Alternative regimen are AZT + 3TC + EFV,AZT + 3TC + NVP and TDF + 3TC + NVP [4, 5].

The first ART regimen offers the best opportunity for effective virological suppression and immune recovery [5, 6]. When there is treatment failure first line drug regimen will be substituted with second line regimen [6]..

Even though ART treatment decrease mortality from HIV/AIDS; occurrence of ART treatment failure is common. As cohort study conducted in 16 Sab-Sahara Africa (SSA) shows about 1·6 people on first line in every 100 patients each year shifted second line ART [7]. Many studies also showed that by 2020, the number of adult patients on second-line ART will be at least double as compared to 2014. By 2030, specially 2 million people in Sab-Sahara Africa (SSA) will be on second-line ART while the range of patients on ART remains constant, but the number (proportion) of patients on second-line ART will be increased to 0·8–4·6 million (6·6%– 19·6%) [8].

Even though Ethiopia uses differentiated ART service delivery system still ART treatment failure is high [9]. Retrospective study conducted in public hospitals in Addis Ababa showed that magnitude of ART treatment failure is 19.8% of which 15, 6.3 and 1.3% is immunological, clinical and virological failure respectively [10]. Systemic review and meta-analysis conducted in Ethiopia 2018 showed that a pooled prevalence of HIV treatment failure was 15.9% and this treatment failure is more common in Oromia regional state (18.8%) than other regional state in Ethiopia and this systemic review also showed poor ART adherence, WHO clinical stage III/IV and presence of opportunistic infections are significantly associated with first line ART treatment failure [11].

Occurrence of first line ART failure has many negative out comes. As the research findings shows, drug resistance will be increased after patient shifted second line ART (NNRTIs65.5%, NRTIs, 53.3%, PIs 1.1%) [12]. Even in developing countries the cost of second line ART is very high, that is second-line antiretroviral therapy (ART) costs 24% more per year than first-line [13]. Drug on regimens of first line are simpler to use, less toxic and more convenient as fixed-dose combinations than second line ART [4].

First-line led to longer life expectancy (28.8 years) and lower life time costs ($41,350/person, from lower second-line costs) [14]. Some studies also showed that the occurrence of un- favorable outcome as death, lost to follow up and virologic failure occurs at rate of 7.9 patients per 100 person years follow-up in patients on second line ART regimens [15]. Many research also revealed that early mortality, drug toxicity and resistance is high in second line ART [16]. Other finding also showed the chance of viral sup- repression is low and death is high after first line ART treatment failure [17]. Therefore, this study was conducted to identify the factor contribute for first line ART treatment failure and identify direction to act on it accordingly to maintain the patient on first line ART for long duration of time.

## Methods

### Study design and setting

Facility based case control study was employed among HIV-infected adults patients on ART at St. Luke referral hospital and Tulubolo general hospital from March 15 to April 222,019. Data was collected from patient himself and record of patients’ cards. St. Luke Referral hospital is located in Wolisso town in south west shoa zone of the Oromia Region, 114 km south west of Addis Ababa the capital site of Ethiopia. In this hospital ART clinic started 2006. It is currently giving ART services for a total of adult 1433 HIV positive patients. Out of those 198 patients are on second line ART while 1235 are on first line ART. Currently St. Luke referral hospital is serving a population in the catchment area of roughly more than 1.3 Million people. This hospital has 200 beds and an outpatient department that sees over 350 patients per day [18]. The total people receiving ART at this hospital is 1563 adult and children.

Tulubolo general hospital is located at direction of southwest from Addis Ababa capital city of Ethiopia. It started to work in 2010. It is located 90 km on the road in the direction of south west from Addis Ababa and it is currently serving 201 patients from which patients on second line ART are 33 and 141 patients are on first line ART. In this hospital the rest 27 patients are with age less than 15 years olds who are being treated in pediatric out-patient department.

### Source and study population

All HIV-infected adult patients in both hospitals who were on ART were source population and all HIV-infected adult patients who were taking ART during the study period at study area were study population. Cases were patients on second line ART while controls were patients on first line ART with age ≥ 15 years old, who were on first line ART for greater than 6 months and who were still on first line ART during data collection period.

Excluded cases were patients on second line ART and who were involuntary to participate; who were having incomplete data and patients on second line ART who transferred in during on second line ART from other health facilities**.**

### Sample size and sampling procedure

**Fig. 1:**
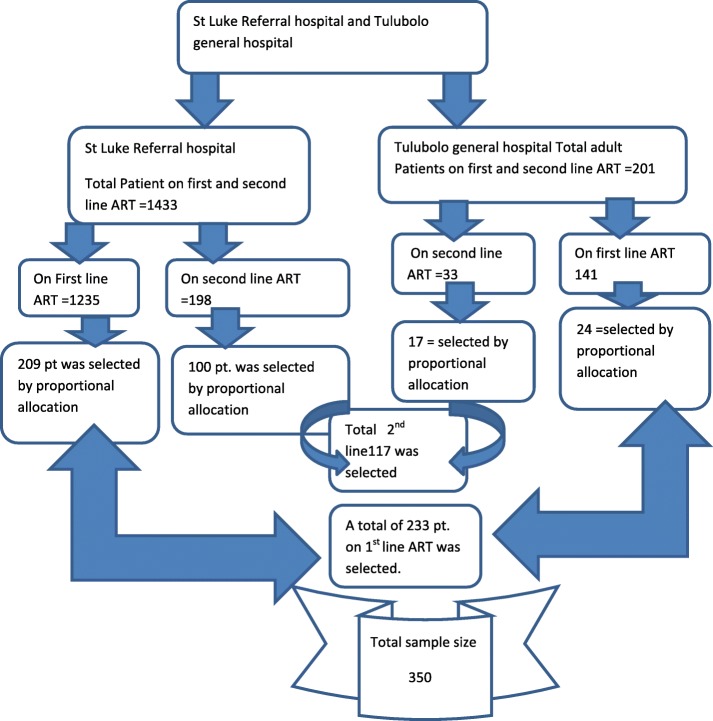
Proportional allocation of patient on ART at St. Luke referral hospitals and Tulubolo general hospital, 2019

Sample size was calculated for the known significantly associated independent variables such, CD4 cell count≤200ceell/mm, BMI < 18.5 g/dl. Then, the largest sample size in most literatures is found to be hg < 11 g/dl. This Hg < 11 g/dl was used to calculate sample size as independent variable. The sample size was calculated using Epi Info version 7 considering the following parameters: As obtained from this previous conducted research proportion of hemoglobin(Hg) < 11 g/dl is 76.1% in cases and 60.99% in controls [19]. By using 5% margin of error, 80% power, a case to control ratio of 1:2 and using a two population proportion formula, the calculated sample size was 350 (117 cases and 233 controls). By using this result proportional allocation were done for both hospitals (Fig. 1).

The sampling procedure employed was systematic sampling techniques. Since those hospitals have the patient schedule first the schedule of the patients who have to go to the hospital within study period was identified. Accordingly; from St. Luke referral hospital 439 patients on first line ART were having schedule to go to the hospitals to take ART in the study period. Then from St. Luke referral hospital to get 209 patients from those who were have the schedule in the study period every second coming patients starting from first coming patient on first line ART was asked. To decide start point from first coming or second coming patients, lottery method was made and the first coming patient was accepted with interval of two. In this hospital again 121 patients on second line ART were having the schedule to go to the hospital in this study period. Then since the number of patients on second line ART were relatively low, to get 100 patients every patient on second line ART were consecutively asked until the sample filled.

From Tulubolo hospital 51 patients on first line ART were having schedule to go to the hospital. To get 24 patients during the study period every second coming patients starting from first coming patient were asked. Again in this Tulubolo referral hospital 24 patients on second line ART were having the schedule to go to the hospital and again for this every individual were asked until the sample of 17 patients on second line ART have obtained.

### Data collection tool and procedure

The data collection tools were developed from federal ministry of health of Ethiopia ART guide line, ART follow up form and patients’ medical cards [4]. Also others tools were adopted from some previous conducted researches [20–23]. The patient information collecting sheet, tools was prepared in English language. The question was having five parts that are Socio-demographic characteristics, clinical characteristics of patients, laboratory measures and ART treatment related conditions, health facilities related questions and behaviors of HIV/AIDS patients those who were on ART. Some questions were obtained from case manager sheets, while the others were obtained from asking the patients.

The data were collected by three BSc nurses from which one at Tulubolo hospital while the remaining for St. Luke referral hospital. There was also one supervisor from one health care worker for each hospital. To get necessary information the medical record number of the patient was reviewed. The data was collected from both the patients and card review. The mandatory question obtained from the patient was first asked and then those from the card at the end. Data were extracted from patient charts/record review/ using a pretested structured checklist and patient himself /herself. Adherence status was the recent record in non-treatment failure and the end of records during patient period on first line ART in treatment failure group. When the incomplete card or involuntary patient was appeared, the next patient was asked. For the cases all question refers, history or record where the patient were on first line ART. This means nothing was asked about when the patient was on second line ART.

Regarding to adherence variables, since the patient shift from first line to second line at different time there is change in definition of virologic failure and immunologic failure. So definition of virologic failure/immunologic failure is also varied from participant to participant and time to time. Therefore, the presence of documented virological failure/immunologic failure at the time of patient record was accepted. This adherence has three categories that is Poor Adherence (< 85%) if the patient miss to take less 6 of 30 prescribed doses or > 9 doses of 60 prescribed dose, fair adherence (85–94%) if the patient miss to take 3–5 of 30 prescribed doses or 3–9 doses of 60 prescribed dose and good adherence (> 90) if the patient miss to take ≤2 of 30 prescribed doses or ≤ 3 doses of 60 prescribed dose [4].

### Data quality assurance and analysis

To maintain data quality, first the question was translated in local language Afan Oromo then it was retranslated back in to English language by other persons to see the consistence of the questions. Training was given for data collectors and supervisor on issue of confidentiality and privacy of data and how to collect the information from patients and the card. The questionnaires were pre-tested before the start of the actual data collection on 5% of sample size at Amaya hospital. Based on the pre-test, question was revised, edited, and those found to be unclear and that question that couldn’t be available was removed.

Supervisor and the principal investigator were supervising the data collection process daily. Data completeness was checked daily by the supervisor and principal investigator.

After checked for completeness data was entered in to Epi data version3 and exported to STATA SE version 14 for analysis. First one new variable were created and named as groups. Then data was entered as 1 = cases and 0 = controls. Data was cleaned and edited by running simple frequencies and cross tabulation before analysis. Media with Standard Deviation (SD) mean with confidence interval and percentages were used to summarize characteristics in each group. Chi-square test was used to identify weather there is association between dependent and independent variables. Bivariable and multivariable logistic regression was used to identify predictors of first line ART treatment failure. Independent variables with *p* ≤ 0.25 in the bivariate analysis were included in the multivariable analysis. Variable in the final model was selected by step-wise backward selection procedure. Model of goodness of fit was done by log likelihood goodness of fit test. Crude and adjusted odds ratio with 95% confidence intervals was computed and statistical significance variables was considered with two sided *P*-value < 0.05.Then the final finding was presented by table, graph and description.

## Result

### Socio-demographic characteristics of participants

A total of 350 participants (117 cases and 233 controls) were participated in the study. The number of female participant was191 (54.57%) of the total participants. The median age for cases participants at enrolment was 34 years (IQR 29, 39) and for controls median age was 34 years (IQR 29, 40). More than half of the participants were married for both case 62(52.99%) and controls groups 127(54.51%). Regarding to residence of the patients more than half of the cases75 (64.1%) and again more than half of the controls 162(69.53%) patients were living in the urban area during on first line ART.

Forty eight (41.03%) cases and one hundred one controls (43.35%) participant’s age at enrolment was between age group twenty five to thirty four. Less than quarter of the cases participants’ 17(14.53%) and 32(13.73%) control participants’ age was with age above forty five. Greater than half of the participant’s religion was orthodox Christianity for both cases76 (64.96%) and controls 146 (62.66%) groups. Regarding to supporting person greater than three fourth of the cases group 93(79.49%) and again greater than three fourth of controls 202(86.7%) groups of participants were having somebody to support them when they were on first line ART (Table 1).

**Table 1:** Socio-demographic characteristics of patients taking ART drugs at St Luke referral hospital and Tulubolo general hospital, 2019

Variable		Cases n (%)	Controls n (%)	Total
Sex	Male	62(52.99)	97(41.63)	159 (45.42)
	Female	55 (47.01)	136(58.37)	191(54.58)
Total		117	133	350(100)
Age	< 25	13(11.11)	22(9.44)	35(10)
	25–34	48 (41.03)	101(43.35)	149(42.57)
	35–44	39 (33.33)	78(33.48)	117(33.43)
	> = 45	17 (14.53)	32(13.73)	49(14)
Total		117(100)	133(100)	350
Ethnicity	Oromo	91(77.78)	167(71.67)	258(73.71)
	Amara	16(13.68)	45(19.31)	61(17.43)
	Others	10(8.55)	21(9.01)	31(8.86)
Total		117(100)	133(100)	350 (100)
Residence	Urban	75(64.1)	162(69.53)	237(67.71)
	Rural	42(35.90	71(30.47)	113(32.29)
Total		117(100)	133(100)	350 (100)
Marital status	Single	15(12.82)	25(10.73)	40 (11.43)
	Married	62(52.99)	127(54.51)	189 (54)
	Separated/divorced	13(11.11)	43(18.45)	56(16)
	Widowed	27(23.08)	38(16.31)	65(18.57)
Total		117(100)	133(100)	350 (100)
Discordance at base line	Positive	53(85.48)	86(67.72)	139(20.63)
	Negative	9(14.52)	41(32.28)	50(26.45)
Total		62	127	189
Education status of the patient	Can’t read and write	37(31.62)	65(27.90)	102(29.14)
	Primary	47(40.17)	87(37.34)	134(38.28)
	Secondary	26(22.22)	59((25.32)	85(24.28)
	College and above	7(5.98)	22(9.44)	29(8.28)
Total		117(100)	133(100)	350 (100)
Occupation	Farmer	34(29.06)10(8.55)	66(28.33)	100(28.57)
	Government worker	9(7.69)	35(15.02)	45(12.85)
	Student	43(36.75)	12(5.15)	21(6)
	Merchant	12(10.26	73(31.33)	116(33.14)
	Laborer	9(7.69)	32(13.73)	44(12.57)
	Others		15(6.44)	24(6.85)
Total		117(100)	233(100)	350 (100)
Support	Yes	93(79.49)	202(86.7)	295(84.28)
	No	24(20.51)	31(13.3)	55(17.72)
Total		117(100)	233(100)	350 (100)

(Other ethnicity Guraghe, Tigray walayita, Kebene. Other occupation, driver, carpenter, tailor, police)

### Clinical characteristics of participants

During base line ART initiation fifty two of the case participants (44.44%) were classified in WHO clinical stage II and nearly half of controls groups 111 (47.64%) were classified in WHO clinical stage I. Regarding presence or absence of base line opportunistic infection greater than three- fourth of the cases have base line opportunistic infection at the time of ART initiation 92 (78.63%) and greater than half of the controls have base line opportunistic infection 123 (52.79%).Tuberculosis co infection was occurred in less than quarter of both groups that was in17 (12.82%) of cases and 12(5.15%) of controls (Table 2).

**Table 2:** Clinical characteristics of cases and controls of patients on ART at St Luke referral hospital and Tulubolo general hospital, 2019

Variable		Case n (%)	Controls n (%)	Total
Base line functional status	Working	102(87.18)	227(97.42)	329(94)
		15(12.82)	6(2.58)	21(6)
Total		117(100)	233(100)	350 (100)
Baseline WHO stage of HIV	Stage I	24(20.52)	111(47.64)	135(38.57)
	Stage II	52(44.44)	84(36.05)	136(38.850
	Stage III/IV	41(35.04)	38(16.31)	79(22.57)
Total		117(100)	233(100)	350 (100)
Base line body mass index	<=16	8(6.84)	11(4.72)	19(5.42)
	16.01–18.5	48(41.03)	74(31.76)	122(34.85)
	> 18.5	61(52.14)	148(63.52)	209(59.71)
Total		117(100)	233(100)	350 (100)
Recent BMI	<=16	4(3.42)	4(1.72)	8 (2.28)
	16.01–18.5	12(10.26)	17(7.30)	22(6.28)
	> 18.5	101(86.32)	212(90.99)	213(60.85)
Total		117(100)	233(100)	350 (100)
Base line opportunistic infection	Yes	92(78.63)	123(52,79)	215(61.43)
	No	25(21.37)	110 (47.21)	135(38.57)
Total		117(100)	233(100)	350 (100)
OI after initiate ART	Yes	95(81.20)	148(63.52)	243(69.42)
	No	22(18.80)	85(36.48)	107(30.58)
Total		117(100)	233(100)	350 (100)
TB co infection	Yes	17(14.53)	12(5.15)	29(8.28)
	No	100(85.47)	221(94.85)	321(91.71)
Total		117(100)	233(100)	350 (100)
Chronic non communicable diseases during on first line ART	Yes	15(12.82)	26(11.16)	41(11.72)
	No	102(87.18)	207(88.84)	309(88.28)
Total		117(100)	233(100)	350 (100)
Presence of malnutrition during on first line ART	Yes	21(17.95)	25(10.73)	46(13.15)
	No	96(82.05)	208(89.27)	304(86.85)
Total		117(100)	233(100)	350 (100)
Cotrimoxazole taken	Yes	112(95.73)	187(80.26)	299(85.42)
	No	5(4.27)	46(19.74)	51(14.57)
Total		117(100)	233(100)	350 (100)

### First line ART treatment related condition and laboratory result of the participants

**Fig. 2:**
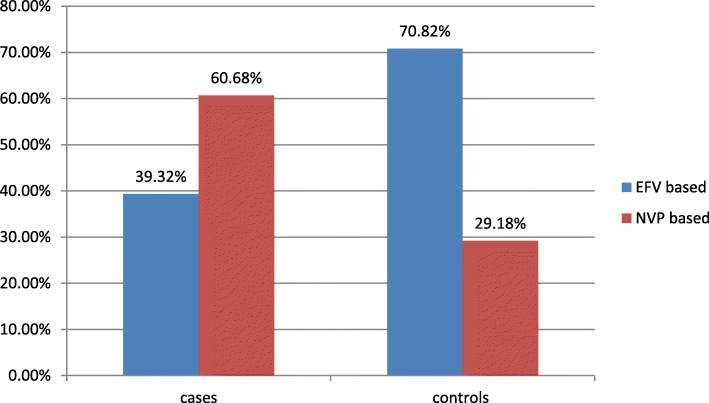
NNRTI based first line ART regimen of the participants at St. Luke referral hospital and Tulu bolo general hospital, 2019

Regarding base line ART NRTI nearly half of cases 49 (41.88%) and greater than half of controls 156 (66.95%) had started with TDF based first line ART. Also forty eight (41.03%) of cases and 44(18.88%) of controls had started base line ART with AZT. Regarding to NNRTI in cases more NVP was used 71 (60.68%) than in controls group 68 (29.18%). EFV was more used in controls group 165 (70.82%) than in cases group 42 (39.32%) as a first line ART (Fig. 2).

**Fig. 3:**
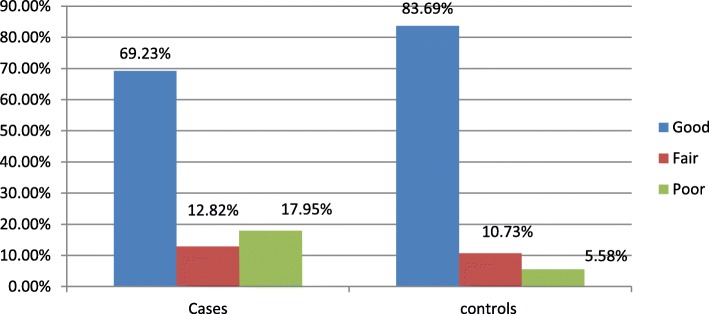
ART adherence status of the participants at St. Luke referral hospital and Tulu bolo general hospital, 2019

Regarding to disclosure status of the patient to family, partner or other else three fourth of cases 91 (77.78%) disclosed their HIV infection status and in the control groups almost the same proportion that is three- fourth of them 178 (76.39%) disclosed their HIV infection status to others. Greater than half of the participants were having history of good adherence status in both cases and controls (69.23 vs83.69%) (Fig. 3).

First line ART drug substitution was more common in patient with first line ART 43(36.75%) than in second line ART 48 (20.60%) and majority of both cases group 97(82.91%) and controls group 219(93.99%) had no history of lost to follow up (Table 3).

**Table 3:** ART treatment related condition of cases and controls of adult patient on antiretroviral therapy at St. Luke Referral Hospital and Tulubolo general hospital, south west shoa Oromia regional state Ethiopia, 2019

Variables		Cases n(%)	Controls n(%)	Total
NRTI baseline	TDF base	49(41.88)	156(66.95)	205(58.58)
	AZT based	48(41.03)	44(18.88)	92(26.28)
	D4Tbased	20(17.09)	33(14.16)	53(15.14)
Total		117(100)	233(233)	350(100)
Lost to follow up	Yes	20(17.09)	14(6.01)	34(9.72)
	No	97(82.91)	219(93.99)	316(90.28)
Total		117(100)	233(233)	350(100)
Disclosure being HIV positive to family/others	Yes	91(77.78)	178(76.39)	269(76.85)
	No	26(22.22)	55(23.61)	81(23.14)
Total		117(100)	233(233)	350(100)
Was there occurrence of drug side effect	Yes	17(14.53)	11(4.72)	28(8)
	No	100(85.47)	222(95.28)	322(92)
Total		117(100)	233(233)	350(100)
Duration on first line ART	< 5 yrs	17(14.53)	1006(42.92)	117(33.42)
	> = 5 yrs	100(85.47)	133(57.08)	233(66.58)
Total		117(100)	233(233)	350(100)
First line ART drug substitution	Yes	43(36.75)	48(20.60)	91(26)
	No	74(63.25)	185(79.40)	259(74)
Total		117(100)	233(233)	350(100)
Time lag to start ART after diagnosed positive	The same month	21(17..95)	71(30.47)	92(26.28)
	1 to 24 month	78(66.67)	146 (62.66)	224(64)
	Greater than 24 month	18(15.38)	16(6.87)	34(9.72)
Total		117(100)	233(233)	350(100)
Baseline CD4 count	< 100	46 (39.32)	8 (3.43)	54(15.42)
	> = 100	71 (60.68)	225 (96.57)	296(84.58)
Total		117(100)	233(233)	350(100)
Recent CD4 count	< 100	20 (17.09)	1(0.0.43)	21(94)
	> = 100	97 (82.91)	232(99.57)	329(94)
Total		117(100)	233(233)	350(100)
Baseline hemoglobin	< 11	22 (18.80)	30(12.88)	52(14.85)
	> = 11	95(81.20)	203(87.12)	298(85.14)
Recent hemoglobin	< 11	5(4.27)	1(0.43)	6(1.71)
	> = 11	112(95.73)	232(99.57)	344(98.29)
Total		117(100)	233(233)	350(100)

During ART initiation forty six (39.32%) of the cases had base line Cd4 less than 100 cell/mm3 while only 8 (3.43%) of controls had base line cd4 less than 100 cell/mm3. Regarding to hemoglobin level during the patients initiation of first line ART greater than three-fourth of the control groups were having base line hemoglobin> 11 g/dl 203(87.12%). Regarding to base line CD4 the median CD4 for case was 128cell/mm3 (IQR 65,243) but for control median CD4 was250 cell/mm3 (IQR 149, 382).

### Health care facility and participants characteristics

In greater than three fourth of cases group 96 (82.05%) and again in greater than three fourth of controls group178 (76.39%) mode of identified HIV infection was by PICT. Having history of post exposure prophylaxis was more common in control 8(3.43%) groups than in cases 2(1.71%). Majority of both cases’ group 102(87.18%) and controls’ group 224 (96.14%) participants were initiated first line ART at the Hospital (Table 4).

**Table 4:** Health care facility and patient related factors of cases and controls for of adult patient on antiretroviral therapy at St. Luke Referral Hospital and Tulubolo general hospital, south west shoa Oromia regional state Ethiopia,2019

Variables		Cases n (%)	Controls n (%)	Total
Health facility where start first line ART	Hospitals	102(87.18)	224(96.14)	326(93.14)
	Health centers	15(12.82)	9(3.86)	24(6.85)
Total		117(100)	233(233)	350(100)
Suspected route of infection	I don’t know	25(21.37)	41(17.6)	66(18.85)
	Sexual intercourse	86(73.5)	178(76.39)	264(75.42)
	Sharp material	6(5.13)	14(6.01)	20(5.71)
Total		117(100)	233(233)	350(100)
Mode of first identified ART	VCT	21 (17.97)	55(23.61)	76(21.71)
	PICT	96 (82.05)	178(76.39)	274(28.28)
Total		117(100)	233(233)	350(100)
History of taking of Post exposure prophylaxis	Yes	2(1.71)	8(3.43)	10(2.85)
	No	115 (98.29)	225(96.57)	340(97.14)
Total		117(100)	233(233)	350(100)
Confirmed criteria for treatment failure	Virological	73(62.39%)		
	Clinical/immunological	14(11.96%)		
	Clinical/ virological	11(9.41%)		
	Immunologic/virological	8(6.83%)		
	All criteria	11(9, 41%)		

### Bivariable and multivariable analysis of predictors of first line ART treatment failure

After so many variables as having history of supporting people or not, WHO clinical stage, base line functional status, tuberculosis co infection, first line ART drug substitution, sex, base line opportunistic infection, opportunistic infection after initiated ART, base line ART, adherence status, base line Cd4,duration on first line ART, time lag to initiate ART after confirmed positive, occurrence of first line ART drug side effect in the patients and having history of malnutrition which have *p* value less than 0.25 were entered into multivariable logistic regression and from those only some variables have been remained into multivariable logistic regression.

From those which were remained into multivariable logistic regression again only a few of them were significantly associated with first line ART treatment failure. Accordingly, the result from multivariable analysis indicated that there was positive significant association between facing first line ART treatment failure and patients having base line opportunistic infection (AOR 2.06, 95%CI 1.12, 3.79). The finding showed the odds of having base line opportunistic infection were 2.06 times higher among case than controls group. There was also significant association between staying on first line ART for greater than 5 years and occurrence of first line ART treatment failure that was the odds of having history of staying on first line ART for greater than 5 years was3.42 times higher among cases than control groups (AOR3.42, 95%CI 1.63, 7.19) (Table 5).

**Table 5:** Multivariable analysis of predictors of first line ART treatment failure among HIV positive adult patients at St. Luke Referral hospital and Tulubolo General Hospital, 2019

Variables		Cases	Control	COR(95% CI)	AOR(95% CI)	*P* value
Sex	Male	62	97	1	1	
	Female	55	136	0.63(.40,.98)	0.65(0.37,1.14)	0.137
OI after ART started.	Yes	95	148	1		
	No	22	85	0.40(0.23,0.68)	0.56(0.28,1.09)	0.089
History of malnutrition	Yes	21	25	1	1	
	No	96	208	0.54(0.29,1.03)	0.55(0.25,1.23)	0.151
Base line opportunistic infection	Yes	92	123	3.29(1.97,5.48)	1.97(1.06,3.63)	0.030*
	No	25	110	1	1	
LFU	Yes	20	14	3.22(1.56,6.65)	3.66(1.44,9.27)	0.006*
	No	97	219	1	1	
Adherence status	Good	81	195	1	1	
	Fair	15	25	1.44(0.72,2.88)	1.48(0.64,343)	0.357
	Poor	21	13	3.88(1.85,8.13)	1.70(0.70,4.12)	0.233
NNRTI ART based	EFV base	46	165	1	1	
	NVP base	71	68	3.74(2.34,5.96)	2.77(1.22,6.28)	0.014*
Duration on First line ART	< 5 yr	17	100	1		
	> = 5 yr	100	133	4.42(2.48,7.86)	3.42(1.63,7.19)	0.001*
Baseline Cd4	< 100	46	32	4.06(2.40,6.88)	2.72(1.46,5.07)	0.002*
	> = 100	71	201	1	1	
Base line ART NRTI	TDF base	49	156	1	1	
	AZ based	48	44	3.47(2.06,5.8)	1.05(0.44,2.50)	0.895
	D4Tbased	20	33	1.92(1.01,3.66)	39(0.13,1.10)	0.770
ART drug toxicity	Yes	17	11	1	1	
	No	100	222	0.29(0.13,.64)	0.42(0.16,1.08)	0.072
Time lag to initiate ART after confirmed HIV positive	In the Same month	21	71	1	1	
	In 1 to 24 month	78	146	1.80(1.03,3.15)	2.14(1.06,4.34)	0.033*
	After 24 month	18	16	3.80(1.65,8.73)	3.82(1.37,10.6)	0.010*
Tuberculosis co-infection (at initial)	Yes	17	12	1	1	
	No	100	221	0.31(0.14,0.69)	0.50(0.20,1.24)	0.138
1st line ART substitution	Yes	43	48	1	1	
	No	74	185	0.44(0.27,0.73	0.59(0.29,1.18)	0.136

The aim of this study was to investigate determinants of first line ART treatment failure and revealed that the odds of having base line opportunistic infection at first line ART initiation were 1.97 times higher among case-patients than controls (AOR 1.97, 95% CI 1.06, 3.63). This implies cases group were 1.97 times more likely had base line opportunistic infection than control groups. Regarding to lost to follow up the Odds of having previous history of lost to follow up found among cases group were 3.66 times higher as compared to controls groups (AOR 3.66, 95%CI 1.44, 9.27). This also showed us that having history of lost to follow up was significantly associated with first line ART treatment failure.

Considering to first line ART regimen the odds of initiated first line ART with NVP (NNRTI) based regimen were 2.77 times higher among cases patient than controls (AOR 2.77, 95% CI 1.22, 6.28). This also implies us starting first line ART with NVP based was significantly associated with first line ART treatment failure. The result also revealed the odds of staying greater than 5 years on first line ART were 3.42 times higher among cases than controls (AOR 3.42 95%CI 1.63,7.19). This in turns indicated that first line ART treatment failure was positively associated with staying on first line ART for greater than 5 years.

At the time of first line ART initiation the odds of having CD4 less than 100 cell/mm3 were 2.72 times higher among cases than controls (AOR 2.72, 95% CI 1.46, 5.07) and this indicated having base line CD4 < 100cell/mm3 was significantly associated with first line ART treatment failure. Again the finding indicated the odds of starting ART after 2 years of confirmed HIV positive were 3.82 times higher among cases patients than controls (AOR 3.82,95%CI 1.37,10.6), while the odds of started ART within 2 to 24 month were 2.14 times more among cases than controls (AOR 2.14,95% CI 1.06,4.34). So delaying ART taking after being confirmed HIV positive was positively associated with bringing occurrence of first line ART treatment failure.

## Discussion

The objective of this study was to identify determinants of first line ART treatment failure. Accordingly, the this study findings revealed that base line opportunistic infection, staying on first line ART for greater than 5 years, starting ART after 2 month of being confirmed HIV positive, Initiate base line ART with NVP based NNRTI, having history of lost to follow up and having base line CD4 less than 100cell/mm3 were identified to be significantly associated with first line ART treatment failure.

Accordingly, presence of opportunistic infection as herpes zoster, bacterial pneumonia, diarrhea, pulmonary and extra pulmonary tuberculosis, PCP, toxoplasmosis and meningococcal infection present at the time of the patient initiate first line ART was identified as significant factors for first line ART treatment failure. This is in line with other studies conducted in Indonesia, India, Woldia Hospital in north part of Ethiopia and Addis Ababa Ethiopia [19, 23–26]. This may be due to the presences of those opportunistic infection further decreases the immunity of the patients.

When the immunity of the patients decreases due to those opportunistic infection and HIV virus itself the chance of the patient to control the amount of the virus in the body is too low and as a result the first regimen of ART can’t control the HIV infections condition. The presences of those opportunistic infections bring a very conducive environment for the HIV virus. Other justification for this is that the patient may encounter burden of two diseases treatment that is HIV /AIDS and the opportunistic infections. Also this may be due to potential interaction between ART drugs and opportunistic infection therapy that makes the ART ineffective and leads first line ART treatment failure. The opportunistic infection present at the time of ART initiation may be not cured and this lead clinical failure.

The result from this study again further identified that the odds of having CD4 less than 100cell/mm3 were higher among case participants than their counter part control participants that was significantly contribute treatment failure. The result showed that having base line CD4 less than 100cee/mm3 was significantly associated with first line ART treatment failure. This study findings was consistence with other studies as determinants of first line ART treatment failure conducted in public hospital in Addis Ababa Ethiopia, Felege Hiwot Referral hospital, Debre Markos Referal hospital in Ethiopia, Zimbabwe, Tanzania,south Africa and China [19, 22, 27–31]. The reason behind this is that the target cells for HIV infection is the CD4 T cells. As the CD4cells decreases this is the chance for opportunistic infection to occurred and lead clinical failure. In another way this low CD4 means high viral load that in turns brings virological failure. This initial low CD4 is difficult to be boosted enough in HIV infected patients that may also lead immunologic failure. This brings management of HIV to be difficult and it needs other regimen of ART to control this HIV infection effect [32].

Including the above determinant factors other finding from this study was that staying on first line ART for greater than 5 years was significantly associated with first line ART treatment failure. The result indicated that the odds of staying on first line ART were higher among cases group than control groups. This finding was in line with other studies conducted in the China,Tanzania University of Gondar referral hospital, Felege Hiwot referral hospital [20, 28, 33, 34]. This may be due to as the time patient stay on first line ART increases severity of HIV/AIDS, drug resistance of the HIV virus, chance of occurrence of opportunistic infection will increase. In addition to this other justification for this is that first line ART treatment failure can be increased due to decreased immunity of the patients as the time goes toward old age. As the time goes the chance of the patients to exposed many factors that have synergic effect for treatment failure is high. Other justification for this is that as the time goes again the chance of occurrence of viral mutation is high and this in turns brings occurrence of first line ART treatment failure.

In addition to what was mentioned in the above this study finding also revealed that Nevirapine based first line ART initiation was significantly associated with increases occurrence of first line ART treatment failure more than EFV based NNRTI. The result indicated that the odds of initiated base line ART with NVP regimen were higher in individual with second line ART than their counter part individual on first line ART. This finding was supported with study conducted in southern Africa [35]. This may be due to the more common side effect occurred in the side of NVP than in EFV. This side effect as rash, nausea, fatigue, headache, vomiting, diarrhea abdominal pain and muscle pain are more common in NVP. This side effect may become difficult for the patients and as a result its adherence may be disturbed and contribute for first line ART treatment failure.

Other explanation for this is that this may be due to EFV is given only one time per day but NVP is twice a day and this may increase chance of adherence disturbance that may contribute for treatment failure. This finding is supported with systemic review and meta-analysis conducted in South Africa [36]. Other research finding also indicated that in patient with opportunistic infection as tuberculosis drug- drug reaction is not more common in EFV based and patient take it without other burden [37].

In addition to what was mentioned in the above this study findings also showed that having history of lost to follow up were more important determinant factors for fist line ART treatment failures. This finding indicated that the odds of having history of lost to follow up were higher among cases than control participant. This finding was consistence with other studies conducted in Kenya and Tanzania [34, 38]. The possible justification for this is that this having history of lost to follow up may bring first line ART treatment failure because of occurrence of drug resistance, increased viral load during lost to follow up and may be also because of occurrence of other opportunistic infection that further deteriorate the immunity of the patients during this lost to follow up period.

The other findings from this study is that the odds of starting ART initiation after 2 years of being confirmed positive was higher among cases as compared with control group and this was significantly related with first line ART treatment failure. But those who started within two to twenty four month after being confirmed positive were also more likely encountered first line ART treatment failure. This finding was in line with study finding in Tanzania [22]. This time increment significance may be due to increased viral load within the patients and due to faced other related disease associated with HIV/AIDS. In another ways there is also lost of interest or misunderstanding purpose of ART in those who start late and they may even didn’t take ART as prescribed.

### Limitation of the study

Since the case and control classification was only based on what was already confirmed by health professionals some patients could had been included in the study as a control group by having manifestation of clinical failure.

## Conclusion

The finding from this study revealed that lower CD4 that is < 100 CD4cell/ul, long duration on first line ART, having history of lost to follow up, presence of base line opportunistic infection, base line nevirapine based NNRTI and start to take ART after 2 years of being confirmed HIV positive are independent predictors of first line ART treatment failure.

Counseling the patient must be continuous by case manager or the health professionals themselves regarding to these identified determinant of first line ART treatment failure.

To those stake holders acting on HIV/AIDS and government another strategy has to drafted and implemented on the side of the patients. Example, health extension workers have to identify those patients in the kebele and frequently monitoring them by going there house. It is better if there is means of giving NVP once a day since this twice day may be difficult for the patient to manage its adherence (there may be chance of missing one dose that may contribute for treatment failure).

It is better if there is media that has a program on ART treatment failure. There must be again more individual home based teaching for all HIV positive patients. Those areas have to give great attention and further researches have to conduct by different study designs, areas and large sample size.

## Data Availability

The datasets analyzed during the current study are available from the corresponding author upon reasonable request.

## Abbreviations

AOR: Adjusted Odds ratio
ART: Antiretroviral therapy
SSA: Sab Saharan Africa
